# Dimethyl 2-nitro­terephthalate

**DOI:** 10.1107/S160053680803465X

**Published:** 2008-10-31

**Authors:** Pei Zou, Min-Hao Xie, Shi-Neng Luo, Ya-Ling Liu, Yong-Jun He

**Affiliations:** a Jiangsu Institute of Nuclear Medicine, Wuxi 214063, People’s Republic of China

## Abstract

In the mol­ecule of the title compound, C_10_H_9_NO_6_, the two ester groups and the nitro group are inclined at 9.2 (2), 123.3 (6) and 135.2 (5)°, respectively to the mean plane of the benzene ring. In the crystal structure, mol­ecules are stacked along the *a* axis, without any π–π inter­actions.  The stacked columns are linked together by non-classical intermolecular interactions of the type C—H⋯O.

## Related literature

For the use of the title compound in the preparation of 2-amino-dimethyl-terephthalic acid, an inter­mediate for dyes, see: Niu *et al.* (2002 ▶). For related structures, see: Brisse & Pérez (1976 ▶); Huang & Liang (2007 ▶).
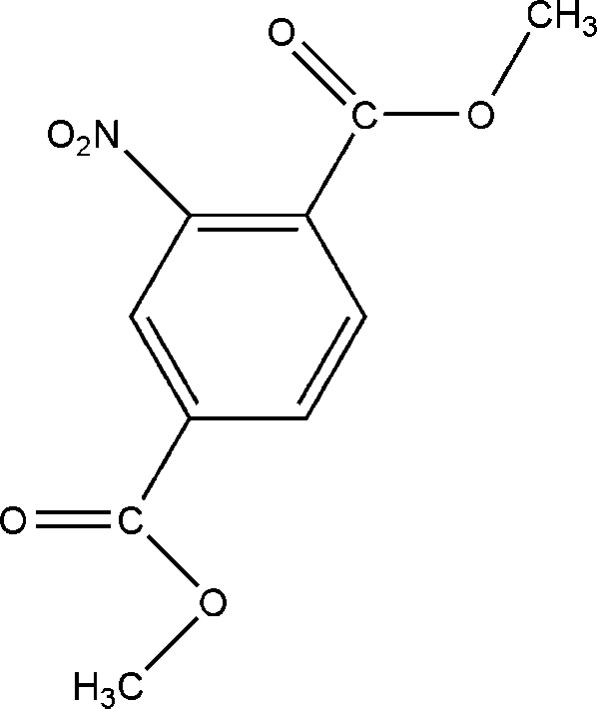


## Experimental

### 

#### Crystal data

C_10_H_9_NO_6_
*M* *_r_* = 239.18
Monoclinic, 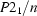

*a* = 6.9080 (14) Å
*b* = 12.662 (3) Å
*c* = 12.231 (2) Å
β = 98.18 (3)°
*V* = 1058.9 (4) Å^3^
*Z* = 4
Mo *K*α radiation
μ = 0.13 mm^−1^
*T* = 293 (2) K
0.30 × 0.30 × 0.10 mm

#### Data collection

Enraf–Nonius CAD-4 diffractometer
Absorption correction: ψ scan (*CAD-4 Software*; Enraf–Nonius, 1989 ▶) *T*_min_ = 0.963, *T*_max_ = 0.987
2052 measured reflections
1889 independent reflections
1245 reflections with *I* > 2σ(*I*)
*R*_int_ = 0.057
3 standard reflections every 200 reflections intensity decay: 2%

#### Refinement

*R*[*F* ^2^ > 2σ(*F* ^2^)] = 0.078
*wR*(*F* ^2^) = 0.201
*S* = 1.00
1889 reflections
156 parameters
H-atom parameters constrained
Δρ_max_ = 0.29 e Å^−3^
Δρ_min_ = −0.31 e Å^−3^

### 

Data collection: *CAD-4 Software* (Enraf–Nonius, 1989 ▶); cell refinement: *CAD-4 Software*; data reduction: *XCAD4* (Harms & Wocadlo, 1995 ▶); program(s) used to solve structure: *SHELXS97* (Sheldrick, 2008 ▶); program(s) used to refine structure: *SHELXL97* (Sheldrick, 2008 ▶); molecular graphics: *SHELXTL* (Sheldrick, 2008 ▶); software used to prepare material for publication: *SHELXL97*.

## Supplementary Material

Crystal structure: contains datablocks I, global. DOI: 10.1107/S160053680803465X/pv2115sup1.cif

Structure factors: contains datablocks I. DOI: 10.1107/S160053680803465X/pv2115Isup2.hkl

Additional supplementary materials:  crystallographic information; 3D view; checkCIF report

## Figures and Tables

**Table 1:** Hydrogen-bond geometry (Å, °)

*D*—H⋯*A*	*D*—H	H⋯*A*	*D*⋯*A*	*D*—H⋯*A*
C1—H1*B*⋯O2^i^	0.96	2.59	3.523 (7)	164
C4—H4*A*⋯O2^ii^	0.93	2.54	3.185 (5)	127

Symmetry codes: (i) 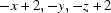; (ii) 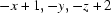.

**Fig. 1.**
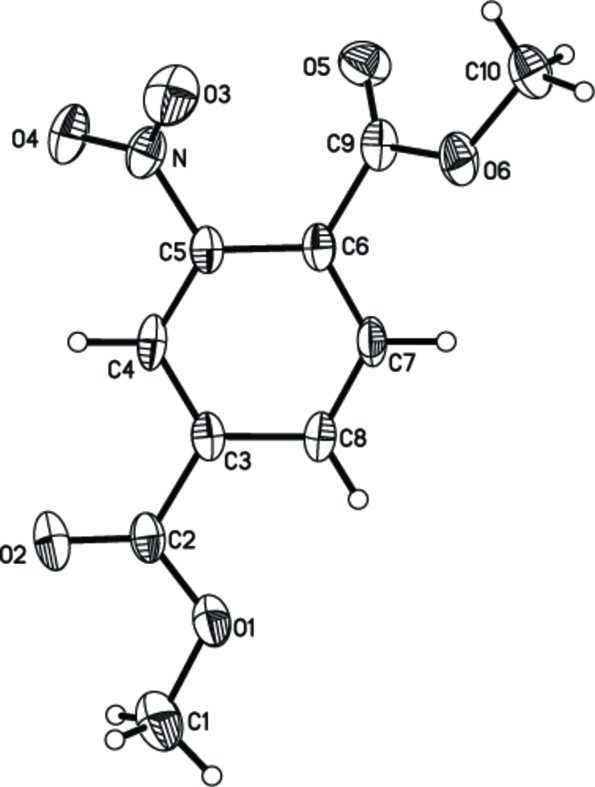
A view of the molecule of (I) with the atomic numbering scheme. Displacement ellipsoids were drawn at the 30% probability level.

## supplementary crystallographic information

### Comment

The title compound, (I), is useful as a raw material for the preparation of 2-amino-dimethyl-terephthalic acid, which is used as an important intermediate for dyes (Niu  *et al.*, 2002). The structures of dimethyl terephthalate (Brisse & Pérez, 1976) and dimethyl 2,3-dihydroxyterephthalate (Huang & Liang, 2007) which are closely related to the title compound have already been reported. In this article, we report the crystal structure of (I). A view of the molecule of (I) is presented in Fig. 1. The bond lengths and angles are within expected ranges. The C1/O1/C2/O2, C10/O6/C9/O5 and O3/N/O4 planes form dihedral angles of 9.2 (2), 123.3 (6) and 135.2 (5)°, respectively, with the C3—C8 plane. In the crystal structure, the molecules are stacked along the *a* axis, without any π-π interactions. The stacked columns are linked together by non-classical intermolecular interactions of the type C—H···O (Table 1).

### Experimental

A sample of commercial 2-nitro-dimethyl-terephthalic acid (Aldrich) was crystalized by slow evaporation of a solution in methanol.

### Refinement

Positional parameters of all the H atoms bonded to C atoms were calculated geometrically and were allowed to ride on the C atoms to which they are bonded, with *H*—*C*(aryl) = 0.93 Å and *U*_iso_(H) = 1.2*U*_eq_(C) or with *H*—*C*(methyl) = 0.96 Å and *U*_iso_(H) = 1.5*U*_eq_(C).

### Crystal data

**Table:**

C_10_H_9_NO_6_	*F*(000) = 496
*M**_r_* = 239.18	*D*_x_ = 1.500 Mg m^−^^3^
Monoclinic, *P*2_1_/*n*	Mo *K*α radiation, λ = 0.71073 Å
Hall symbol: -P 2yn	Cell parameters from 25 reflections
*a* = 6.9080 (14) Å	θ = 10–13°
*b* = 12.662 (3) Å	µ = 0.13 mm^−^^1^
*c* = 12.231 (2) Å	*T* = 293 K
β = 98.18 (3)°	Block, colourless
*V* = 1058.9 (4) Å^3^	0.30 × 0.30 × 0.10 mm
*Z* = 4	

### Data collection

**Table:**

Enraf–Nonius CAD-4 diffractometer	1245 reflections with *I* > 2σ(*I*)
Radiation source: fine-focus sealed tube	*R*_int_ = 0.057
graphite	θ_max_ = 25.3°, θ_min_ = 2.3°
ω/2θ scans	*h* = −8→8
Absorption correction: ψ scan (CAD-4 Software; Enraf–Nonius,1989)	*k* = 0→15
*T*_min_ = 0.963, *T*_max_ = 0.987	*l* = 0→14
2052 measured reflections	3 standard reflections every 200 reflections
1889 independent reflections	intensity decay: 2%

### Refinement

**Table:**

Refinement on *F*^2^	Secondary atom site location: difference Fourier map
Least-squares matrix: full	Hydrogen site location: inferred from neighbouring sites
*R*[*F*^2^ > 2σ(*F*^2^)] = 0.078	H-atom parameters constrained
*wR*(*F*^2^) = 0.201	*w* = 1/[σ^2^(*F*_o_^2^) + (0.05*P*)^2^ + 3.5*P*] where *P* = (*F*_o_^2^ + 2*F*_c_^2^)/3
*S* = 1.00	(Δ/σ)_max_ < 0.001
1889 reflections	Δρ_max_ = 0.29 e Å^−^^3^
156 parameters	Δρ_min_ = −0.31 e Å^−^^3^
0 restraints	Extinction correction: SHELXL97 (Sheldrick, 2008)
Primary atom site location: structure-invariant direct methods	Extinction coefficient: 0.053 (8)

### Special details

**Table:**

Geometry. All e.s.d.'s (except the e.s.d. in the dihedral angle between two l.s. planes) are estimated using the full covariance matrix. The cell e.s.d.'s are taken into account individually in the estimation of e.s.d.'s in distances, angles and torsion angles; correlations between e.s.d.'s in cell parameters are only used when they are defined by crystal symmetry. An approximate (isotropic) treatment of cell e.s.d.'s is used for estimating e.s.d.'s involving l.s. planes.
Refinement. Refinement of *F*^2^ against ALL reflections. The weighted *R*-factor *wR* and goodness of fit *S* are based on *F*^2^, conventional *R*-factors *R* are based on *F*, with *F* set to zero for negative *F*^2^. The threshold expression of *F*^2^ > σ(*F*^2^) is used only for calculating *R*-factors(gt) *etc*. and is not relevant to the choice of reflections for refinement. *R*-factors based on *F*^2^ are statistically about twice as large as those based on *F*, and *R*- factors based on ALL data will be even larger.

### Fractional atomic coordinates and isotropic or equivalent isotropic displacement parameters (Å^2^)

**Table:**

	*x*	*y*	*z*	*U*_iso_*/*U*_eq_	
O6	0.0321 (5)	0.4552 (3)	0.8800 (3)	0.0576 (9)	
O2	0.7697 (5)	0.0539 (2)	1.0189 (3)	0.0634 (10)	
O1	0.8957 (5)	0.2101 (3)	1.0787 (3)	0.0584 (9)	
O5	0.0234 (5)	0.3733 (3)	0.7173 (3)	0.0633 (10)	
O4	0.1497 (6)	0.0806 (3)	0.7385 (3)	0.0755 (12)	
N	0.1105 (6)	0.1519 (3)	0.8006 (3)	0.0545 (11)	
C3	0.5871 (6)	0.2093 (3)	0.9698 (3)	0.0397 (10)	
O3	−0.0550 (5)	0.1733 (3)	0.8156 (4)	0.0806 (13)	
C2	0.7594 (7)	0.1483 (3)	1.0248 (4)	0.0445 (11)	
C8	0.5802 (7)	0.3193 (3)	0.9806 (4)	0.0489 (11)	
H8A	0.6838	0.3561	1.0201	0.059*	
C6	0.2593 (6)	0.3208 (3)	0.8712 (3)	0.0412 (10)	
C7	0.4154 (7)	0.3717 (3)	0.9309 (4)	0.0516 (12)	
H7A	0.4098	0.4447	0.9382	0.062*	
C9	0.0911 (7)	0.3822 (3)	0.8120 (4)	0.0470 (11)	
C10	−0.1157 (8)	0.5271 (4)	0.8335 (5)	0.0629 (14)	
H10A	−0.1305	0.5819	0.8859	0.094*	
H10B	−0.2373	0.4901	0.8155	0.094*	
H10C	−0.0790	0.5579	0.7677	0.094*	
C5	0.2728 (6)	0.2107 (3)	0.8613 (3)	0.0386 (10)	
C4	0.4337 (7)	0.1556 (3)	0.9090 (4)	0.0465 (11)	
H4A	0.4398	0.0827	0.9006	0.056*	
C1	1.0681 (8)	0.1571 (5)	1.1324 (5)	0.0769 (17)	
H1A	1.1662	0.2086	1.1583	0.115*	
H1B	1.1174	0.1106	1.0808	0.115*	
H1C	1.0357	0.1170	1.1939	0.115*	

### Atomic displacement parameters (Å^2^)

**Table:**

	*U*^11^	*U*^22^	*U*^33^	*U*^12^	*U*^13^	*U*^23^
O6	0.073 (2)	0.0433 (18)	0.060 (2)	0.0163 (17)	0.0214 (17)	−0.0090 (16)
O2	0.075 (2)	0.0335 (18)	0.087 (3)	0.0090 (17)	0.0289 (19)	0.0070 (17)
O1	0.062 (2)	0.0383 (18)	0.076 (2)	0.0080 (16)	0.0165 (18)	0.0019 (17)
O5	0.065 (2)	0.075 (3)	0.049 (2)	0.0151 (19)	0.0045 (17)	−0.0057 (18)
O4	0.099 (3)	0.048 (2)	0.081 (3)	−0.005 (2)	0.017 (2)	−0.028 (2)
N	0.063 (3)	0.042 (2)	0.061 (3)	−0.017 (2)	0.019 (2)	−0.001 (2)
C3	0.051 (3)	0.029 (2)	0.044 (2)	0.0019 (19)	0.0248 (19)	0.0009 (18)
O3	0.052 (2)	0.083 (3)	0.113 (3)	−0.017 (2)	0.036 (2)	−0.010 (3)
C2	0.054 (3)	0.032 (2)	0.053 (3)	0.001 (2)	0.025 (2)	0.004 (2)
C8	0.064 (3)	0.027 (2)	0.058 (3)	−0.002 (2)	0.015 (2)	−0.005 (2)
C6	0.053 (3)	0.029 (2)	0.045 (2)	0.0025 (19)	0.020 (2)	−0.0016 (19)
C7	0.071 (3)	0.023 (2)	0.061 (3)	0.006 (2)	0.010 (2)	−0.004 (2)
C9	0.059 (3)	0.036 (2)	0.052 (3)	−0.002 (2)	0.025 (2)	−0.002 (2)
C10	0.068 (3)	0.052 (3)	0.071 (3)	0.018 (3)	0.021 (3)	0.002 (3)
C5	0.048 (2)	0.030 (2)	0.043 (2)	0.0010 (19)	0.0230 (19)	−0.0007 (18)
C4	0.059 (3)	0.023 (2)	0.065 (3)	−0.007 (2)	0.035 (2)	−0.005 (2)
C1	0.076 (4)	0.059 (4)	0.099 (5)	0.016 (3)	0.026 (3)	0.006 (3)

### Geometric parameters (Å, °)

**Table:**

O6—C9	1.345 (5)	C8—H8A	0.9300
O6—C10	1.425 (6)	C6—C7	1.373 (6)
O2—C2	1.200 (5)	C6—C5	1.404 (6)
O1—C2	1.325 (5)	C6—C9	1.497 (6)
O1—C1	1.442 (6)	C7—H7A	0.9300
O5—C9	1.191 (5)	C10—H10A	0.9600
O4—N	1.234 (5)	C10—H10B	0.9600
N—O3	1.214 (5)	C10—H10C	0.9600
N—C5	1.458 (6)	C5—C4	1.371 (6)
C3—C4	1.383 (6)	C4—H4A	0.9300
C3—C8	1.401 (6)	C1—H1A	0.9600
C3—C2	1.496 (6)	C1—H1B	0.9600
C8—C7	1.381 (6)	C1—H1C	0.9600
			
C9—O6—C10	117.2 (4)	O5—C9—C6	126.3 (4)
C2—O1—C1	115.7 (4)	O6—C9—C6	109.9 (4)
O3—N—O4	123.5 (4)	O6—C10—H10A	109.5
O3—N—C5	118.7 (4)	O6—C10—H10B	109.5
O4—N—C5	117.8 (4)	H10A—C10—H10B	109.5
C4—C3—C8	120.4 (4)	O6—C10—H10C	109.5
C4—C3—C2	119.2 (4)	H10A—C10—H10C	109.5
C8—C3—C2	120.5 (4)	H10B—C10—H10C	109.5
O2—C2—O1	125.0 (4)	C4—C5—C6	121.9 (4)
O2—C2—C3	122.5 (4)	C4—C5—N	118.4 (4)
O1—C2—C3	112.5 (4)	C6—C5—N	119.7 (4)
C7—C8—C3	118.3 (4)	C5—C4—C3	119.4 (4)
C7—C8—H8A	120.9	C5—C4—H4A	120.3
C3—C8—H8A	120.9	C3—C4—H4A	120.3
C7—C6—C5	117.1 (4)	O1—C1—H1A	109.5
C7—C6—C9	120.7 (4)	O1—C1—H1B	109.5
C5—C6—C9	122.0 (4)	H1A—C1—H1B	109.5
C6—C7—C8	123.0 (4)	O1—C1—H1C	109.5
C6—C7—H7A	118.5	H1A—C1—H1C	109.5
C8—C7—H7A	118.5	H1B—C1—H1C	109.5
O5—C9—O6	123.7 (5)		
			
C1—O1—C2—O2	0.6 (7)	C7—C6—C9—O6	−48.0 (5)
C1—O1—C2—C3	−179.0 (4)	C5—C6—C9—O6	137.2 (4)
C4—C3—C2—O2	−0.5 (6)	C7—C6—C5—C4	0.1 (6)
C8—C3—C2—O2	178.9 (4)	C9—C6—C5—C4	175.1 (4)
C4—C3—C2—O1	179.1 (4)	C7—C6—C5—N	179.0 (4)
C8—C3—C2—O1	−1.4 (6)	C9—C6—C5—N	−6.1 (6)
C4—C3—C8—C7	1.3 (7)	O3—N—C5—C4	135.0 (5)
C2—C3—C8—C7	−178.2 (4)	O4—N—C5—C4	−43.2 (6)
C5—C6—C7—C8	−0.3 (7)	O3—N—C5—C6	−43.9 (6)
C9—C6—C7—C8	−175.3 (4)	O4—N—C5—C6	138.0 (4)
C3—C8—C7—C6	−0.4 (7)	C6—C5—C4—C3	0.7 (6)
C10—O6—C9—O5	−1.9 (7)	N—C5—C4—C3	−178.1 (4)
C10—O6—C9—C6	174.5 (4)	C8—C3—C4—C5	−1.5 (6)
C7—C6—C9—O5	128.2 (5)	C2—C3—C4—C5	178.0 (4)
C5—C6—C9—O5	−46.5 (7)		

### Hydrogen-bond geometry (Å, °)

**Table:**

*D*—H···*A*	*D*—H	H···*A*	*D*···*A*	*D*—H···*A*
C1—H1B···O2^i^	0.96	2.59	3.523 (7)	164
C4—H4A···O2^ii^	0.93	2.54	3.185 (5)	127

Symmetry codes: (i) −*x*+2, −*y*, −*z*+2; (ii) −*x*+1, −*y*, −*z*+2.
